# Notes and Comments

**Published:** 1923-05

**Authors:** 


### NOTES AND COMMENTS.

### UNTAXED CIDER.

UNTIL Budget Day everything that the Englishman drinks was taxed, including even patent medicines and water. In some places, indeed, the water rate is distinctly high, while the stamp on bottles of medicine which the doctor does not prescribe produces results that are grateful and comforting to the Chancellor of the Exchequer. Happily Mr. Baldwin has seen his way to the liberation of cider and perry from the clutches of the Excise, and the duty of 4d. a gallon on those beverages which was imposed to help pay for the war has been taken off. The consumption of cider has greatly increased of late years, thanks to improved methods of manufacture and the planting of better stocks, and we may now expect a still further growth of its popularity. Cider is a peculiarly healthy and refreshing drink, and, as a rule, its alcoholic content is so small that it can hardly be described as a "hard" drink. The cyclist, who needs little to eat, but needs, or thinks he does, a good deal to drink, often finds, indeed, that the wayside innkeeper treats it as non-alcoholic and provides it at hours when more lethal beverages are unprocurable. The devoted cider-drinker knows that age cannot wither nor custom stale its infinite variety, since no two barrels were ever alike. This may not be scientific, but it is often delightfully surprising. The vexed question of the medicinal effects of cider has not yet been settled, and probably it never will be, since very often more depends upon the constitution of the drinker than upon the properties of the drink. But that many gouty and rheumatic subjects find that it holds their enemy at bay is beyond doubt. If "an apple a day keeps the doctor away," it is not unreasonable to suppose that a daily bottle of cider may have the same effect. But it is essential that it should be cider and not some "fizzy" decoction full of sugar and chemicals.

### THE INTOXICATING LIQUOR BILL.

THIS matter of cider cropped up again in the Standing Committee of the House of Commons which has been considering Lady Astor's Intoxicating Liquor Bill. By an amendment, reluctantly accepted by Lady Astor, any person over sixteen may order beer, porter, cider, or perry with a meal, provided the meal is not consumed in the bar. We see no objection whatever to a boy of sixteen drinking cider, which is in an entirely different position from beer and stout, and there is clearly some hardship in not allowing a youth on a country walk or a cycling run to drink a glass of cider unless he eats with it. The real solution of the difficulty would be to take draught cider out of the category of alcoholic drinks. The Bill has undoubtedly been weakened by the new clause which provides that a licencee shall not "knowingly" sell intoxicants to be consumed on the premises to persons under eighteen. This gives the publican a rather large loophole. Yet, even in its modified form, the Bill will be of real value. It is an instalment of the many reforms which our public-houses need. What we await now is that some enterprising and public-spirited firm, such as Messrs. Lyons, should show us the way to the public-house of the future—a house of refreshment and recreation rather than a drinking shop.

### ABOLISHING COCAINE.

IT is already clear that international action is the only effective means of controlling the illicit sale of cocaine and other deleterious drugs; but, so far as cocaine is concerned, Sir W. M. Bayliss and Dr. Saleeby have been suggesting that we should go a long step further and entirely abolish its use. It is no longer needed in dentistry, completely effective substitutes, such as novocaine, being available; moreover, the new synthetic substance, "butyn," appears to have established itself, and, like novocaine, it has no action on the central nervous system. If cocaine is no longer of value for legitimate purposes, there is no reason for continuing its manufacture, and so conniving at its abuse. The Opium Committee of the League of Nations is an instrument ready to hand whereby it might be abolished, and although neither America nor Germany are members of the League, they are members of the Opium Committee, which is what really matters in this relation. No single country can hope to prevent the secret importation of this terrible drug; but what no nation by itself can do the League ought to be able to accomplish.

### DISCOMFITED GOTHS.

CROYDON, apparently, is the place where Goths most do congregate. For more than three centuries it has possessed in the Whitgift Hospital a beautiful and interesting building which it was not only ready, but anxious and eager, to demolish on grounds of imaginary convenience. Whitgift is not a hospital according to the modern understanding of the word—it is an *asile* for helpless age in the midst of modern turmoil, and its fellows are to be found in no inconsiderable number scattered about in our old towns. It is a building within which broods the sense of ancient peace. It has been little if at all altered, and life beneath its picturesque and venerable roof is lived very much after the fashion of the early seventeenth century days in which it was raised by the pious benevolence of Archbishop Whitgift, who, as the successor of St. Augustine in the Primatial See, spent much of his time in the archepiscopal palace at Croydon. Had its demolition been demanded by imperative necessity we might have bowed to the inevitable, but the necessity exists no longer, if it ever did. A by-pass road mainly for the use of through motor traffic is being made, partly with public money provided by the taxpayers of Northumberland and Cornwall as well as Croydon, and the congestion of quick traffic which might otherwise have found the Hospital an obstacle will soon be removed or diverted. There was thus no vital reason for its demolition, and the House of Lords, with absolute unanimity, has refused to give the Croydon Corporation the power of destruction for which it asked. Rarely indeed has either House of Parliament passed so unqualified a condemnation upon any municipal body, and we hope that the lesson will be taken to heart by other towns—for Croydon was united in supporting its official Goths—who imagine that hooting motor cars are more important than haunts of ancient peace.

### THE HOSPITAL SATURDAY FUND.

FIFTY years have elapsed since the London Hospital Saturday Fund was instituted by certain far-seeing pioneers of reform in hospital finance. The process of reform is still going on—there can, indeed, be no certainty that it will ever come to an end, even were it desirable that it should. As the needs of hospitals increase and the area which they benefit expands new methods of raising money and extensions of old ones will become necessary, since it would be difficult to imagine any direction of philanthropic effort in which the world can less afford to stand still. The Hospital Saturday Fund was a *devancier* in the systematising of this department of finance, and it has shown itself to be of continuing virility and open to the reception of new ideas. It is now celebrating its jubilee, and it is much to be able to say that in the course of its history it has raised more than a million of money for the London hospitals, nearly one-fifth of which came in last year. It is true that the previous year produced more, but we have been passing through a period of industrial depression unexampled in living memory. There are now many other Hospital Saturday Funds which have done for some of the great provincial towns what the London Fund has helped to do for the capital. The co-ordination of effort for hospital maintenance is one of the great works of the immediate future, which makes it the more important that the existing methods should be kept, like the Hospital Saturday Fund, at a high point of efficienc.y

### CANCER IN HUSBAND AND WIFE.

CANCER being deplorably common, it is inevitable that husband and wife should often die of this disease. It is more significant when both husband and wife die of cancer beginning in the same organ, the tongue or stomach for example. But primary cancer being common in both these organs, we should still expect husband and wife occasionally to develop it in the same organs even if the disease is not contagious or infectious. Now, it is very rare for cancer to begin within the chest, as a primary disease of the lungs or pleuræ. So when both husband and wife develop primary cancer in these structures, it is natural that the coincidence should again raise the question, is cancer *à deux* incidental only, or has the disease been conveyed from one partner to the other ? At a recent session of the Northern Congress for Internal Medicine at Helsingfors, a speaker reported a case of primary cancer of the pleura in husband and wife, the interval between the two cases being nine years. It was argued from these cases and from certain observations made in the course of recent experimental research that cancer *à deux* may be due to infection of one partner by the other, even when there is an interval of many years between the deaths of husband and wife. The speaker, Dr. A. Josefson, suggested that it might be advisable to proceed on the same radical lines with regard to cancer as are already followed in the case of tuberculosis. He would have cancer patients isolated, and their bedding and dwellings disinfected when the patients no longer had use for them. There is much to be said for the latter course ; disinfecting the bedding and dwelling after a patient's death might with advantage be made a routine practice, whatever the alleged cause of death may be. But considering how flimsy is the evidence at present available with regard to the infectiousness of cancer, isolation of the patient must be regarded as a cruel measure wholly unjustified. There are so many diseases for which segregation is recommended as a sovereign remedy that were the craving of every segregationist to be gratified, there would soon be an unsegregated wistful minority pining away in the loneliness of bodily and mental perfection.

### REPLANTING THE BLACK COUNTRY.

WE rejoice to see that the illustrated article on this subject which we published in our April issue has directed fresh attention to the admirable work of the Midland Reafforesting Association, the headquarters of which are at Birmingham. The *Times* has published an interesting account, by its Birmingham correspondent, of what has been accomplished which, if it adds little to our own account, yet brings the subject before a wider audience than a monthly publication can hope to reach. We learn that some 14,000 acres of the Black Country are ready for planting with trees, but that means are lacking, the Association having to depend upon voluntary help. It cannot be pretended that unsightly heaps of shale and slag are conducive either to the physical or the moral health of those who are compelled to live in their midst, and every additional tree that is planted helps to purify the air and to restore natural beauty to districts from which the needs of commerce have banished it. The replanting of spoil-banks has, moreover, an economic side. The plantations that have been made, relatively small though they are, are constantly increasing in value as saleable timber, and in the ever-growing dearth of the world's timber supply, each new plantation adds potentially to the tangible wealth of the country.

### THE HOSPITAL ALMONER.

THE Council of the Institute of Hospital Almoners, whose Annual Report was recently issued, are of opinion that more women are needed for this work if future vacancies are not to remain unfilled. The training extends over a period of two years, and as it is clearly desirable that students whose duties will lie in the provinces should have experience of local conditions, arrangements have been made with the Universities of Birmingham, Bristol, Glasgow, Leeds, Liverpool and the Armstrong College at Newcastle, whereby they can take a considerable part of the training qualifying for an Almoner's certificate in those towns. A portion of the period of training under an Almoner can also be spent at certain hospitals in special areas. The Institute points out that exceptional qualities are required—it is obvious that without a strong interest in humanity and plenty of tact no Almoner would be of value to the Institution with which she is connected. But is it justifiable for any hospital in these days of financial stress to spend money on an Almoner's department? This is a question raised in the report itself, and, in our opinion, there is only one answer. The Almoner is the link between the hospital and the home, and she can do a work of untold value in ensuring that the expenditure on an individual patient is not wasted by a return to unfavourable conditions capable of improvement. As a social worker the Almoner should have a future of ever-widening usefulness. The President of the Institute is Sir Napier Burnett, and the report pays just tribute to the fine work of the late Sir Charles Loch, which led to the movement of social service in hospitals and resulted in the first appointment of Almoners.

### ORGANISED SAFETY WORK.

IT has been as difficult to make the average citizen realise that accidents are not just "incident to his calling" as it was to overcome the superstitions of dark ages which called most epidemic diseases an evidence of some higher displeasure. Accidents are not only mostly avoidable, but very commonly actual blunders, and the remarkable diminution of their frequency which may be achieved by organised safety work is well instanced from the records of American railways. It is not so long ago that the United States was notorious for railway fatalities. Safety organisation has recently, however, been "boomed" there to such good purpose that in one small area alone, where in 1918 the total of injured people was nearly 2,800, last year it was less than 800. The point to note is that this reduction has been brought about not by safety devices but almost entirely by safety education. As an instance of rapid results this is excellent, and the figures might well have been better were it possible to teach everyone, and break the careless habits of the middle-aged and elderly. The American deals with the rising generation in the schools.

### A COATED TONGUE.

MOST of us have always looked upon a furred and coated tongue as being the result of "liver," indigestion and so forth. There was, in all probability, little enough reason for doing so, and we cannot help thinking that there may be more than a grain of truth in the contention that this furry coating is the cause, not the result, of these troubles. In a recent number of the *New York Medicine Journal*, Dr. T. R. French remarks that this covering of the "dirty tongue" consists of the same bacteria and the same materials as are found in septic, inflamed tonsils—a fact with which bacteriologists will certainly agree. He has tested his theory by carefully removing the coating from the tongue, and, without using any other treatment, claims to have noted the rapid disappearance of many of the symptoms and signs in dyspeptics, rheumatics and others. In sickness and in health we should realise that it is just as important to keep the surface of the tongue clean as to perform the same rite for other parts of our body. Brushing the top of the tongue with a tooth-brush after brushing the teeth is as necessary as cleaning the teeth themselves.

### BEGINNING AT THE BEGINNING.

NO great importance need, as a rule, be attached to the pronouncements of amateur committees on matters of morality and sex—such bodies are the happy hunting grounds of all manner of faddists. But there is little need to quarrel with the conclusions in the report on "Youth and the Race" which has just been presented by the Birth-Rate Commission to the Minister of Health. Health and chastity are very closely associated, as this country has had grievous reason to discover since the facts about venereal disease have, so to speak, been placarded on every hoarding, and, although the Commission has nothing really new to say on the subject, what it does say is excellent. More immediately to the point is the Commission's emphatic declaration as to the value of maternity and child welfare centres. That more advantage might be taken of the opportunities these centres afford every medical man will agree, and we are glad to find the Commission condemning the short-sighted agitation for the limitation or weakening of institutions which become more necessary every day. If ever we are to have a really healthy country, which means a country in which preventable disease finds it difficult to get a foothold, we must begin at the beginning, and these centres are a powerful aid to doing so. When people are grown up much of the mischief has been done. In presenting this report to Mr. Neville Chamberlain, the Birth-Rate Commission is preaching to the converted; but there are still vast masses of people outside the Ministry of Health who are in grievous need of grace on this subject.

### THE ULTIMATE RESULTS OF SANATORIUM TREATMENT.

IT is a deplorable fact that the reputation of sanatorium treatment is much better on the Continent than with us. In this country there has been comparatively little sifting of cases suitable for sanatorium treatment. Politics of a kind have swept into English sanatoria many patients with only a few months to live, and when the miraculous failed to happen sanatorium treatment was given the blame. What can be achieved by this treatment has lately been demonstrated by a German doctor, Professor F. Reiche, of Hamburg. In the 10-year period 1895-1904 he examined 2,864 insured consumptives, and found that 1,866 were suitable for sanatorium treatment. In several cases this was repeated once or twice. In 1921 he investigated the subsequent fate of these patients, and, thanks to the methodical organisation of the insurance machinery, he was able to trace the whole of them. It was found that after an average interval of 22 years from the time of discharge, 46·9 per cent. of these patients were fit for work, 35·2 per cent. were dead, and 18·2 per cent. were invalided. In the case of the male patients, 48 per cent., and in the case of the female patients 69 per cent., fitness for part or whole-time work was retained. It should be noted that a goodly proportion of the deaths was due to non-tuberculous causes, and that the Great War accounted for many deaths among combatant ex-sanatorium patients. Professor Reiche made one other interesting observation. He classified his patients according to whether there was or was not a family history of tuberculosis, and he found that the outlook was equally good for the two classes. It follows that when a person develops tuberculosis his outlook is no worse even if his parents and all his brothers, sisters, cousins and aunts have died of the disease.

### DANGEROUS DRUGS.

THE provisions of the Dangerous Drugs and Poisons (Amendment) Bill, which has now passed the House of Commons, constitute, as we said last month, a valuable addition to the legislation on the subject. It will be generally recognised that the provision in Section 1, empowering a Justice of the Peace to grant a search warrant in the case of suspected premises, and also the provisions with regard to penalties, are essential if the detestable traffiickers in these drugs are to be dealt with effectively. The penalties operate not only in regard to offences committed in Great Britain under the laws in force in this country, but also in regard to offences punishable under the provisions of any corresponding law outside Great Britain. A person guilty of an offence against the Act is made liable in respect of each offence on conviction on indictment to a fine not exceeding £1,000, or to penal servitude for a period not exceeding ten years, or both ; or, on summary conviction, to a fine not exceeding £250, or to imprisonment for a term not exceeding twelve months, or to both, as well as in every case to the forfeiture of the goods. These are salutary penalties, and are properly distinguished in the Act from minor penalties for lesser offences in regard to forms and prescriptions which might be committed by doctors or chemists through inadvertence. The Home Secretary showed himself during the passage of the Bill through the House fully alive to the need for the protection of members of the medical profession from any provisions of a meddlesome or inquisitorial character. He promised in particular to annul a regulation made under the earlier Act which prevented medical men from prescribing certain drugs for themselves, and thereby to meet the legitimate complaint that the medical profession as a whole were being unfairly embarrassed for the delinquencies of a very small percentage of their number.

### THE DIPHTHERITIC PENHOLDER.

THE researches of Surgeon-Commander Sheldon Dudley, R.N., into the causes of outbreaks of diphtheria and scarlet fever in the Royal Naval School at Greenwich produced some remarkably illuminating results. There were more than 300 cases of the one disease or the other among the boarders, but every day-boy escaped. One of those minute inquiries which enable the modern doctor to hunt down disease produced a simple explanation of the mysterious immunity of the *externs*. The boarders used a common stock of penholders ; the day-boys brought their own. Certain penholders in a classroom box were found to be heavily infected by virulent diphtheria bacilli. Light was also thrown upon the remarkable circumstance that the boarders did not infect the day-boys ; but that does not so much concern us at the moment. The result of Surgeon-Commander Dudley's investigations is of importance to a far larger circle than that of schoolmasters and schoolboys. Grown-up people are, perhaps, less likely to suck penholders than schoolchildren, yet they very often do it—even the " littery gent " has been known to suck the end of his penholder while searching for the *mot juste*. Hotels, clubs, ocean liners and many other places of public resort keep a common stock of penholders, which it is clearly unwise to suck. Why thought is stimulated by this process is not clear—perhaps some person of a scientific turn of mind will give a little attention to this curious fact.

### AMERICAN HOSPITALS.

AN inquiry into the American hospital system which has just been concluded shows that 57 per cent. of the hospital beds in the City of New York have been endowed by private munificence. This is a noble record ; but America is a very rich country in which the duty of hospital maintenance has been taken closely to heart by those who have money to spend upon benevolence. America is large as well as rich—its population approaches three times that of the United Kingdom—and contains a vast number of hospitals, many of them splendidly equipped. In fact, the inquiry to which we have referred shows that, now that the demands created by the war are much reduced, there are far too many of them. How best to deal with these surplus institutions has suggested to the Commission of Consultants which has just reported the desirability of federating the hospital system of the United States. It is thought that some of the supernumerary buildings provided by the Federal Government to meet war needs might be used as homes for disabled soldiers and as sailors' hospitals, or handed over to improve the health equipment of the States in which they stand. The commission recommends that a Government Hospitals Department should be established which should be a centre of advice on planning, equipment, and cognate matters. In a vast country like the United States help of this kind is perhaps best given from a Federal source—co-ordination might otherwise be exceedingly difficult. In England we are justifiably nervous about Government interference with hospitals, and prefer to keep their management apart altogether from officialdom. The American proposals will, nevertheless, be watched here with interest.

